# How to Promote the Health of the Dentist

**Published:** 1867-04

**Authors:** 


### "HOW TO PROMOTE THE HEALTH OF THE DENTIST."

*Editors Dental Register:*—Dentists as well as physicians generally know enough already as to the laws of health, but it certainly is surprising that men whose chief study is the human system, its diseases, and how to treat them, should be so utterly reckless of the laws of health as many medical men are.

In our specialty there are a few dangers to which we are particularly exposed. One is the vitiated atmosphere that we often have to work in, owing to improper ventilation, and the close contact we are obliged to have with our patients.

Another is the position we are obliged to assume in operating at the chair; and a third is the habit that some Dentists have of frequently going without their regular meals, and over working when business is pressing.

Now it seems hardly worth while to write upon this subject, and yet it is one of vital importance. No Dentist needs to be told of the necessity of proper ventilation; we all understand it, and yet how many of us are poisoned to death and never think of it *till it is too late!*

As to the position we have to assume at the chair, that would do us no injury if we were content to limit ourselves to a proper length of time per day to work, and not try to make up time one day that we have lost another. If we ever lose time we *can't make it up, and it is no use to try.*

And, by the way, Dentists need all the leisure time they can have to study, and post up, and experiment, and when we have much leisure time, it is generally pretty good evidence that we need it, and need to improve it.

As to going without one's dinner, and then working till a late hour before tea as some of us do sometimes, and bent up over the chair at that, what shall I say? The men that do, call themselves fools, (not to be bettered,) and still they do it.

I have only to say, that when I was a student and time and again witnessed my instructor thus *abuse* himself, I resolved, as for me, *give me my regular meals, and especially my dinner*, or let *me starve;* and for ten years I have never failed to have my *dinner*, business or no business.

Gentlemen, a good stomach and good health are something worth having and *keeping*. Let us *care* for those bodies, the health of which is of such priceless value. S.

